# National Sanitation

**Published:** 1905-08-26

**Authors:** 


### National Sanitation.

We are much inclined to the conclusion that the gospel of sanitation, like that of theology, has not always been preached in the right way; and that the shortcomings of the preachers may possibly not be without their effect in the production of a certain indifference of assent to precepts, unaccompanied by any manifest intention of carrying them into practice. Sir James Crichton Browne is reported to have caused great searchings of heart in some quarters by declaring at a recent meeting of the Association of Sanitary Inspectors that the clergy would be better occupied in the practical promotion of cleanliness than in preaching what he is said to have described as "silly sermons"; but we doubt whether the ordinary sanitary sermon is either much better or much wiser than the ordinary clerical one; and it is certainly worth while to inquire why it is that mankind, as a rule, appears to derive so little practical benefit from either form of discourse. The sentiment of the Northern Farmer about the preacher—"An' I thowt a said whot a owt to a' said an' I coom'd awaäy"—is very much that of the average Town Councillor or Poor-law Guardian towards Sir James Crichton Browne and his fellow labourers upon the platform. Their statistics are convincing, their arguments are unassailable, but they somehow fail to produce an impression of reality upon the majority of audiences. Everyone professes to wish for health and longevity; everyone, as a rule, neglects the first principles of sanitation as soon as ever these come into collision with accustomed habits, or resists them as soon as they threaten to curtail accustomed indulgences. It would be thought, for example, that the body of people who constitute what calls itself "Society" would be certain to desire not only good health for the enjoyment of their own advantages, but also the transmission of vigorous constitutions to the children by whom those advantages will be inherited; but the facts of life in any great capital can hardly be said to sustain the supposition. "Let us eat and drink, for to-morrow we die," is as much a principle of action in the present day as it was in the time of the apostle; and to-morrow comes, bringing its tale of premature deaths along with it. Can it be from sheer want of imagination that so many "respectable" people fail to realise the consequences of the customs in which they indulge every day, and, in the words of Sir James Crichton Browne, "sit with hands crossed waiting for the golden age to be conferred by some government or commission."

We referred last week to the frequent failure of county and borough councils, and other so-called authorities, to make any attempt at the fulfilment of their duties in relation to public health; and these failures can only be due to an entire absence of belief in very traceable connections between causes and events. Nothing is more easy of demonstration than that many sanitary defects are certain, soon or late, to entail upon the localities permitting them a larger expense than that of correcting them in the first instance. They always have done so, and they always will. The average councillor either does not realise this, or he hopes that the price ultimately payable for sins of omission will not be exacted while he is in office, and that he may not only escape unpopularity for encouraging expenditure, but also, when the inevitable result comes, may escape condemnation for having rendered the expenditure inevitable by his neglect. Surely there must be something wrong, or at least something infelicitous, in the lines on which the general sanitary teaching of our time has been developed. Perhaps, on the whole, Charles Kingsley was more successful than anyone who either preceded or has followed him; and we would venture to suggest his example as one which the clergy generally might with advantage try to follow. We will not risk giving offence by any endorsement of Sir James Crichton Browne's condemnatory description of sermons in general; but it is certain that it would be possible to write on the duty of preserving both private and public health a long series which, if not convincing, would at least not incur the risk of being justly described as "silly."

